# Genome-Wide Profiling of Pluripotent Cells Reveals a Unique Molecular Signature of Human Embryonic Germ Cells

**DOI:** 10.1371/journal.pone.0039088

**Published:** 2012-06-21

**Authors:** Nikta Pashai, Haiping Hao, Angelo All, Siddharth Gupta, Raghothama Chaerkady, Alejandro De Los Angeles, John D. Gearhart, Candace L. Kerr

**Affiliations:** 1 Department of Biomedical Engineering, Johns Hopkins University School of Medicine, Baltimore, Maryland, United States of America; 2 Deep Sequencing and Microarray Core, High Throughput Biology Center, Johns Hopkins University, Baltimore, Maryland, United States of America; 3 Department of Neurology, Johns Hopkins University, Baltimore, Maryland, United States of America; 4 Stem Cell Program, Institute for Cell Engineering, Johns Hopkins University, Baltimore, Maryland, United States of America; 5 Department of Cell and Developmental Biology, Institute of Regenerative Medicine, University of Pennsylvania, Philadelphia, Pennsylvania, United States of America; 6 Department of Animal Biology, Institute of Regenerative Medicine, University of Pennsylvania, Philadelphia, Pennsylvania, United States of America; 7 Department of Gynecology and Obstetrics, Institute for Cell Engineering, Johns Hopkins University, Baltimore, Maryland, United States of America; 8 Department of Biological Chemistry, Johns Hopkins University School of Medicine, Baltimore, Maryland, United States of America; 9 Stem Cell Transplantation Program, Division of Pediatric Hematology Oncology, Children’s Hospital Boston, Massachusetts, United States of America; 10 Department of Biological Chemistry and Molecular Pharmacology, Dana-Farber Cancer Institute, Harvard Medical School, Boston, Massachusetts, United States of America; 11 Harvard Stem Cell Institute, Cambridge, Massachusetts, United States of America; University of Newcastle upon Tyne, United Kingdom

## Abstract

Human embryonic germ cells (**EGCs**) provide a powerful model for identifying molecules involved in the pluripotent state when compared to their progenitors, primordial germ cells (**PGCs**), and other pluripotent stem cells. Microarray and Principal Component Analysis (**PCA**) reveals for the first time that human EGCs possess a transcription profile distinct from PGCs and other pluripotent stem cells. Validation with qRT-PCR confirms that human EGCs and PGCs express many pluripotency-associated genes but with quantifiable differences compared to pluripotent embryonic stem cells (**ESCs**), induced pluripotent stem cells (**IPSCs**), and embryonal carcinoma cells (**ECCs**). Analyses also identified a number of target genes that may be potentially associated with their unique pluripotent states. These include *IPO7*, *MED7, RBM26, HSPD1*, and *KRAS* which were upregulated in EGCs along with other pluripotent stem cells when compared to PGCs. Other potential target genes were also found which may contribute toward a primed ESC-like state. These genes were exclusively up-regulated in ESCs, IPSCs and ECCs including *PARP1, CCNE1, CDK6, AURKA, MAD2L1, CCNG1*, and *CCNB1* which are involved in cell cycle regulation, cellular metabolism and DNA repair and replication. Gene classification analysis also confirmed that the distinguishing feature of EGCs compared to ESCs, ECCs, and IPSCs lies primarily in their genetic contribution to cellular metabolism, cell cycle, and cell adhesion. In contrast, several genes were found upregulated in PGCs which may help distinguish their unipotent state including *HBA1, DMRT1, SPANXA1, and EHD2.* Together, these findings provide the first glimpse into a unique genomic signature of human germ cells and pluripotent stem cells and provide genes potentially involved in defining different states of germ-line pluripotency.

## Introduction

Primordial germ cells (**PGCs**) are unipotent progenitors of sperm and egg which retain an innate ability to generate pluripotent stem cells *in vivo*, called embryonal carcinomas (**ECCs**), and *in vitro*, known as embryonic germ cells (**EGCs**). It is unknown whether mechanisms similar to those involved in the generation of these cells are also involved in maintaining the pluripotent status of other stem cells such as embryonic stem cells (**ESCs**) and induced pluripotent stem cells (**IPSCs**). In this study, the first genome wide assessments of human PGCs and EGCs were performed and compared with other pluripotent ESCs, IPSCs and ECCs.

PGCs are unipotent in that they are lineage-restricted to become germ cells. They do not exhibit self-renewal and do not survive past one week under standard tissue culture conditions [1]. In the mouse, it is known that PGCs are derived from a region of the epiblast that mainly gives rise to the extra-embryonic mesoderm. In humans, PGCs first appear between the third and fourth week post-fertilization in the endoderm of the dorsal wall of the yolk sac, near the allantois, then proceed to migrate through the hindgut during the fourth week and dorsal mesentery in the fifth week to reach the genital ridge [2]–[4].

Under appropriate conditions, human PGCs can generate pluripotent stem cells. EGCs are pluripotent stem cells derived from PGCs in cell culture using specific growth factors and media formulations. The term EGCs, was given by Donovan *et al.* [5], [6] who derived them from mouse PGCs, in order to distinguish them from ESCs. Since then, a handful of other laboratories have also reported the generation of human EGC lines [7]–[11]. ECC lines are another source of pluripotent cells that are cultured from adult teratocarcinomas from which there is genetic, immunological, and morphological evidence suggesting a PGC origin [12]. Lastly, IPSCs can be generated from PGCs by lentiviral transduction of pluripotent regulators similar to those used to generate IPSCs from somatic cells [13]–[18].

EGCs share the general properties of pluripotent stem cells including unlimited self-renewal and the ability to give rise to cells that represent all three embryonic germ layers. In contrast, EGCs are unlike any other pluripotent stem cell as they are derived from differentiated cells without IPSC-like targeted genetic manipulations and unlike ECCs, maintain a stable karyotype. Furthermore, while human EGCs can generate a variety of cell-types representative of the three germ layers *in vitro*, they do not form teratomas *in vivo* like their mouse EGC counterparts. Therefore, EGCs may exist in a uniquely, partial, or intermediate pluripotent state. As such, comparisons between EGCs and PGCs with other pluripotent stem cells provide a powerful model to identify factors that are associated with different states of pluripotency.

Distinct states of pluripotency have been revealed by several laboratories which have shown that pluripotent stem cells exhibit differences in their clonal or self-renewing and differentiating capacities [19]–[22]. For instance, mouse ESCs and IPSCs in the “naïve state” demonstrate single cell clonal ability, rounded colony morphology, and are not dependent on FGF2 and TGFβ/Activin signaling. In contrast, conventional human ESCs and IPSCs and mouse epiblast-derived stem cells exist in a “primed state” of pluripotency exhibiting flattened colony morphology, insufficient clonal expansion, and a dependence on FGF2 and TGFβ/Activin signaling. These differences in pluripotent states have been attributed to species differences as well as the developmental state of the stem cell origin and yet they are inter-convertible depending on the cell culture environment. For instance, the primed state of human ESCs and IPSCs was shown to be convertible to the naïve mouse ESC-like state given the appropriate factors in cell culture [22]. It has also been shown that mouse EGCs will behave similar to the naïve state of mouse ESCs under similar culture conditions [23]. However, it remains unknown whether human EGCs could also be converted to a naive state. Indeed, there is considerable interest in deciphering the range of multiple pluripotent states in human cells as they could be utilized to partition out mechanisms that regulate distinct attributes of the pluripotent phenotype.

Currently, the pluripotent state of conventional human EGCs is unknown. For instance, like human ESCs, conventional human EGCs express SSEA3, SSEA4 and TRA antigens, TRA-1-60 and TRA-1–80, which are inefficient at clonal expansion and require FGF2 in cell culture [8], [24]. However, similar to mouse ESCs, human EGCs share rounded morphology, express SSEA1 and require LIF for their survival. Given that human EGCs share features in common with both mouse ESCs and human ESCs, it is likely that conventional EGCs fall in their own unique state of pluripotency. Therefore, the following study provides new insight into this question and reveals the genomic signature of EGCs which will identify new candidate genes for regulating pluripotency.

Comparisons between EGCs and PGCs will also help establish a unique signature of human PGCs which have not been demonstrated before while also providing further insight into whether ESCs originate from a PGC origin. Indeed, several lines of evidence suggest that PGCs and ESCs may originate from an early germ cell progenitor [25]–[27]. For instance, several reports have demonstrated that mouse ESCs express genes associated with immature male and female germ cells such as *Stella*, *deleted in azoospermia-like (* ***Dazl*** *)* and *Fragilis* [28], [29]. Similarly, one study that examined the differentiation of human ESCs into germ cells [30], detected the expression of eight genes characteristic of early germ cells in ESCs and none from six genes expressed by later germ cells. Most significantly, this study demonstrated gene expression of *DAZL* by ESCs but not by human inner cell mass (**ICM**). In a recent study, Scholer *et al*. [31] demonstrated significant similarities in gene profiles between mouse PGCs and ESCs. Despite these relevant studies, it is largely unknown how human PGCs resemble ESCs at the molecular level compared to other PGC-derived stem cells.

Global expression analyses have been extensively performed to examine the molecular signature of human pluripotent stem cells. These studies have characterized gene expression profiles of human ESC, IPSC, and ECC lines using microarray and other high-throughput analyses in attempts to identify candidates involved in self-renewal or pluripotency [32]–[37]. In addition to comparisons with mouse stem cells, a meta-analysis performed by Assou et al., [38] provides further statistical significance to genes that have been found enriched in ESCs by these studies. Importantly, the meta analysis revealed the ability of the microarray studies to consistently reveal patterns of similarities and differences in gene-expression patterns among human ESC lines including high levels of *OCT4*, *SOX2,* and *NANOG* expression in human ESCs, three factors which are critical for maintaining pluripotency in mouse [39]–[41] and human stem cells [42]. Other genes associated with pluripotency are also found including *UTF1, REX1, FOXD3* and members of the FGF2, MAPK-ERK, TGFB/activin/nodal, Wnt/b-catenin and Akt/PkB signaling pathways. Together this data demonstrates the efficacy of genomic profiling for determining the “stem-cell” orchestra and provide a foundation for further comparisons of these cells with PGCs and EGCs. Two other studies have also reported on the genomic profiles of PGCs for the purpose of identifying mouse unipotent PGC specific genes that would distinguish them from mouse ESCs [31], [43]. Scholer and colleagues [31] revealed 4 new unipotent PGC specific genes that are highly conserved in mouse and human while Mise *et al*. [43] demonstrated that mouse EGCs were more similar to ESCs compared to mouse PGCs and adult germ line derived stem cells.

In this study, we conduct the first genome-wide comparison study of human PGCs and EGCs with a number of diverse pluripotent stem cell types including ESCs, ECCs, and IPSCs. Genomic analyses classified these cell types into three distinct groups, 1) PGCs, 2.) EGCs, and 3.) IPSCs, ESCs ECCs, that reflected their developmental potential. These comparisons included qualitative and quantitative differences in known pluripotent associated gene expression as well as revealing a novel signature for human EGCs. Thus, these findings provide important information for identifying potential mechanisms required for PGC reprogramming into the pluripotent state. Comparisons between human PGCs and ESCs will also help facilitate the identification of factors associated with germ line development and may help establish factors related to a common germ cell progenitor potentially shared between these cell types.

## Results

**Figure 1:**
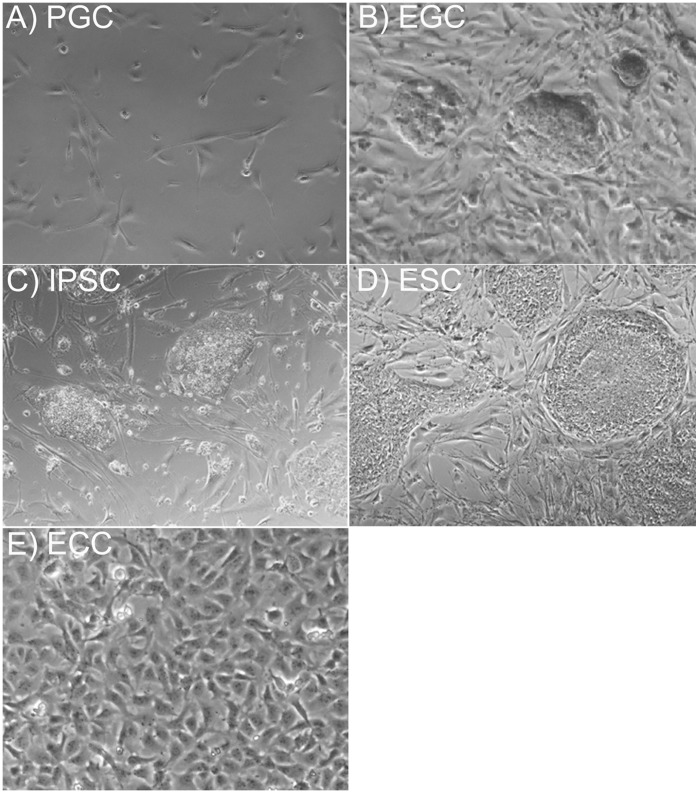
Phase contrast images representing different stem cells studied in gene expression analyses. (A) primordial germ cells (PGCs), (B) embryonic germ cells (EGCs), (C) induced pluripotent stem cells (IPSCs), (D) embryonic stem cells (ESCs), and (E) embryonal carcinoma cells (ECCs).

Gene expression was studied in five different cell types including PGCs, EGCs, ECCs, IPSCs, and ESCs (**Figure 1**). In contrast to PGCs, which were directly isolated from tissue, cell lines analyzed in this study were isolated between passage 15 to 27. ECC and ESC lines were XY, while PGCs, EGCs, and IPSCs represented both XX and XY genotypes. PGCs were diploid at this stage in development as previously described [24], [44]. Both EGC and IPSC lines were derived from SSEA1+ sorted PGCs and IPSCs generated using lentiviral integration of *SOX2, OCT4* and *MYCN* genes into PGCs. Details of the cells used in this study are described in **Table S2** **in Supplementary Material**.

### Quantitative RT-PCR Validation of known Pluripotent and Germ Cell Associated Genes in PGCs, EGCs and Pluripotent Stem Cells

**Figure 2:**
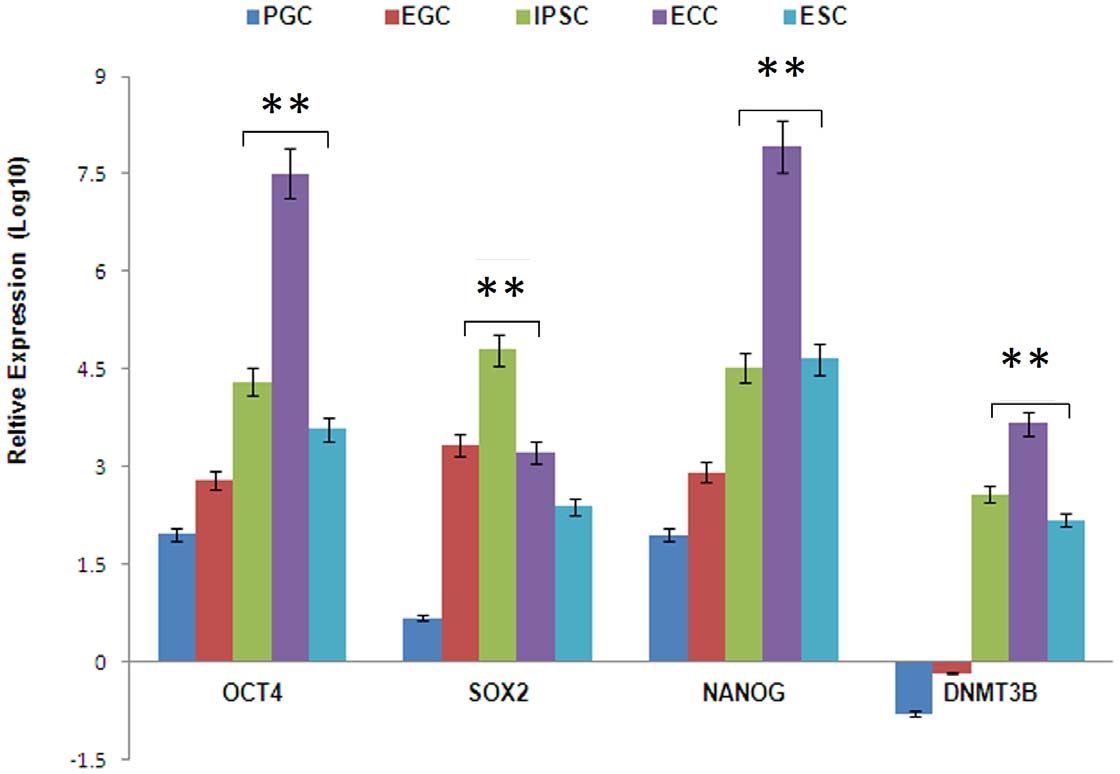
Real-time qRT-PCR analysis of several key pluripotent and germ cell associated genes in primordial germ cells and pluripotent stem cells. *OCT4, SOX2, NANOG, and DNMT3B* were normalized to the *beta-actin* gene using the comparative CT method, and plotted relative to the human foreskin fibroblast line, HFF1 (0 baseline) (N  = 3 biological samples with technical triplicates for each cell type, P<0.05). Asterisks denote statistical significant differences in cell lines compared to PGCs.

Real-time quantitative RT-PCR analysis (**Figure 2**) was used to validate the expression of genes known as unique signatures of germ cells and the pluripotent state in our study populations. This was performed on 3 independent cell lines from each group different from those analyzed by microarray analysis. **Figure 2** shows data for *OCT4, SOX2, NANOG,* and *DNMT3B* from 2–3 independent biological specimens or cell cultures as in the case of ECCs, ESCs, and a human foreskin-derived fibroblast line, **HFF1**, and each experiment was performed in triplicate. HFFs were used to study relative expression across all groups. Results showed that PGCs expressed higher levels of all pluripotent stem cell genes compared to fibroblasts except *DNMT3B*. Furthermore, the expression of all pluripotent genes was considerably higher in ESCs, IPSCs, and ECCs compared to the PGCs. Likewise, EGCs expressed elevated levels of *SOX2* similar to ESCs, IPSCs, and ECCs. In fact, this significant increase in *SOX2* compared to PGCs in addition to the slightly elevated levels of *OCT4* and *NANOG* expression in EGCs is the most distinguishing feature between these two populations. Although protein levels were not measured in this study, it has been previously reported and shown in our observations that the SOX2 protein is not significantly detectable in human PGCs, unlike mouse PGCs [45]. Therefore, the results of qRT-PCR suggests that the regulation of the SOX2 protein in human PGCs is at the transcriptional level and that regulators of *SOX2* gene expression may play a significant role in PGC reprogramming to EGCs. These results are also consistent with microarray and hierarchical analyses which distinguish *SOX2* expression as a unique signature of the ESC, IPSC, and ECC group compared to PGCs. Another interesting observation was the down regulation of *DNMT3B* in both PGCs and EGCs compared to ESCs, IPSCs, and ECCs. This is consistent with the role of *DNMT3B* in DNA methylation for defining pluripotency in human cells.

### Pair-wise Comparisons of Gene Expression Profiles of PGCs and Pluripotent Stem Cell Lines

To explore the similarities in gene expression profiles, pair-wise comparisons and Pearson’s correlations were performed (**Table 1** **)**. As expected, populations within the same cell types revealed the strongest correlations in gene expression patterns ranging from 0.85–0.99. Variability within groups was the least with IPSCs or ECCs lines which showed the highest correlations within their respective groups at 0.99, P<0.001 each, while PGCs were 0.96 and EGCs ranged from 0.85–0.94. Several possibilities may contribute to the variation found in and among these stem cell lines including the contribution of sex-linked genes and by the passage of time in culture. For instance, when different types of pluripotent stem cell lines are compared, the highest correlations are seen between ECC lines and ESCs (ranging from 0.93–0.94). These lines are both XY and similar in subculture passages. However, ECCs are distinct in their tumorigenic properties from other ESC-like stem cells in that they are malignant cancer stem cells. Nonetheless, the malignant nature of ECCs is not a major contributing factor underlying the close association seen between ESCs. Likewise, it is clear that IPSCs though comprised of both sexes and of an earlier passage than ESCs and ECCs are more similar to male ECCs (0.90–0.91) and ESCs (0.86–0.88) than to either PGCs (.78–.83) or EGCs (.77–.89). This data is consistent with the pluripotent nature of the IPSCs. Thus, it appears that while sex and subculture passages may contribute to some of the differences seen across cell lines, the close associations found among these cells can be primarily contributed by their pluripotent state.

**Table 1:** Pair-wise comparisons of gene expression profiles.

	EGC1	EGC2	EGC3	ESC	PGC1	PGC2	ECC1	ECC2	IPSC1	IPSC2	IPSC3
**EGC1**		0.8997	0.8987	0.8327	0.8931	0.8560	0.8494	0.8469	0.8855	0.8846	0.8698
**EGC2**	0.8469		0.9399	0.8034	0.9174	0.8975	0.7709	0.7671	0.7887	0.7897	0.7669
**EGC3**	0.8987	0.9399		0.8295	0.9091	0.8858	0.8011	0.7983	0.8076	0.8091	0.7892
**ESC**	0.8327	0.8034	0.8295		0.8731	0.8372	0.9356	0.9346	0.8685	0.8712	0.8779
**PGC1**	0.8931	0.9174	0.9091	0.8731		0.9606	0.8363	0.8338	0.8313	0.8349	0.8220
**PGC2**	0.8560	0.8975	0.8858	0.8372	0.9606		0.7983	0.7958	0.7927	0.7962	0.7829
**ECC1**	0.8494	0.7709	0.8011	0.9356	0.8363	0.7983		0.9949	0.8993	0.8997	0.9133
**ECC2**	0.8469	0.7671	0.7983	0.9346	0.8338	0.7958	0.9949		0.8977	0.8992	0.9138
**IPSC1**	0.8855	0.7887	0.8076	0.8685	0.8313	0.7927	0.8993	0.8977		0.9958	0.9746
**IPSC2**	0.8846	0.7897	0.8091	0.8712	0.8349	0.7962	0.8997	0.8992	0.9958		0.9747
**IPSC3**	0.8698	0.7669	0.7892	0.8779	0.8220	0.7829	0.9133	0.9138	0.9746	0.9747	

The expression profiles of EGCs shows the highest similarity to PGCs (∼0.89) compared to ECCs, IPSCs, and ESCs (averaging between 0.80–0.82). This is expected as EGCs are derived from PGCs. While the IPSCs and ECCs were also derived by PGCs, it has been hypothesized that ESCs are also derived from an early PGCs progenitor present in the inner cell mass. These results suggest the EGCs may represent a distinct state of pluripotency consistent with differences seen between EGCs and other pluripotent stem cells grown under traditional culturing methods. For instance, human EGCs, derived under these conditions are more unstable, in that they spontaneously differentiate in culture. EGCs also do not form teratomas in immunocompromised mice even though they can generate a variety of cell types *in vitro*. Thus, the expression profile of EGCs compared to their unipotent progenitor and other pluripotent stem cells may be indicative of the EGCs unique pluripotent state. Additionally, it would be important to delineate the fraction of genes that may be affected by these cell culturing attributes. Such examination could benefit from the comparisons performed herein among these cell types.

### Multivariate Comparisons of Gene Expression Profiles of PGCs and Pluripotent Stem Cells

**Figure 3:**
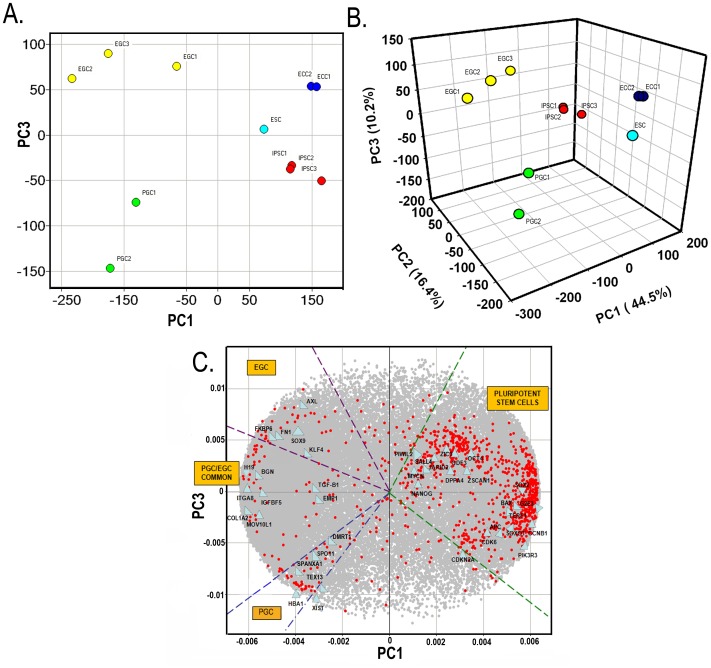
Principal component analysis (PCA) of the expression profiles of primordial germ cells and pluripotent stem cells. (A) Two-dimensional PCA map of cell types, comparing PC1 and PC3. (B) Three-dimensional map comparing all cell types. (C) A loading scatter plot for the identification of signature genes. Red dots represent the top 1000 differentially genes expressed across the mean of all lines with FDR adjusted P<0.0001. “PGC signature” genes include *HBA1, TEX13,* and *SPO11* located on the lower left half of the scatter plot. In contrast, XIST was upregulated in PGCs compared to other pluripotent stem cell lines as expected given their differentiated state. “PGC/EGC common” genes include *H19, BGN, ITGA8, IGFB5, MOV10L1*, and *TGF-B1*. “EGC signature” genes include *SOX9, KLF4, FN1, AXL,* and *FKBP6*. “ECC, ESC, and IPSC” signature genes include *DPPA4, NANOG, SOX2, PIWIL2, MYCN, GDF3,* and *OCT4.*

Global gene expression patterns of PGCs, EGCs, ECCs, IPSCs, and ESCs were analyzed using PCA, which reduces redundancies in variability within high dimensional array data into a smaller number of principal components. Data for PCA including log ratio values and P-values are included in **Table S3**. **Figure 3A** shows the plotted position of each cell population against the PC1 and PC3 axes and **Figure 3B** is representative of the PC1, PC2, and PC3 axes in three dimensional (3D) space. All three PCs accounted for about 71% of the variation present in the entire data set (PC1; 0.445, PC2; .164, PC3; .102). From **Figure 3A** a distinguishable grouping difference can be seen between ESCs, ECCs, and IPSCs which are grouped together and distinctly located away from EGCs and PGCs along the PC1 and PC3 axis. For instance, PGCs are located in the bottom left side of the PCA plot while ECCs, IPSCs, and ESCs are shifted toward the right along the PC1 component axis and EGCs are grouped in the upper left corner along the PC3 axis. Thus, most of the gene expression differences (44.5%) accounted for were along the PC1 axis and may be to the developmental state of these cells. There was also a greater difference along the PC3 axis between EGCs and PGCs compared to ESCs, ECCs, and IPSCs located in between. Thus, the genes associated with the PC3 axis (constituting 10% of the variation) are likely to define differences in the intermediate pluripotent state of EGCs that are independent of the primed human ESC-like state. **Figure 3B** is a three-dimensional plot representing signature genes of each cell type and correlates with **Figure 3A** in terms of their location. Together, these results identify three distinguishing groups of cells which comprise 1.) unipotent PGCs, 2.) EGCs, and 3.) primed human ESC-like stem cells encompassing ECCs, IPSCs and ESCs.

Next, we tried to detect genes that define the characteristics of the pluripotent stem cell and PGC groups based on the PCA results. Because PGCs are well separated along the PC3 axis, genes that make a large contribution to PC3 were sought using a loading scatter plot, shown as **Figure 3C**. Grey dots represent genes which were not in the top differentially expressed genes shown in red. Genes represented by red circles in **Figure 3C** were the most highly differentially expressed genes (1000 of 54,000, or 1.85% genes in the array) (**Table S4**) compared to the overall mean across all cell lines with FDR adjusted P<0.0001. The PGC group samples were located between lines at angles of 3.75 and 4.05 radians on the 2D PCA plot (**Figure 3A**), and genes mapped in the corresponding space by blue lines in the loading scatter plot (**Figure 3C** **)** showed elevated expression in the PGC group. These signature genes show enhanced expression in PGCs compared to other cell groups. Genes plotted between 2.10 and 2.80 radians (purple lines in **Figure 3C**) represent “EGCs signature” genes. Green dotted lines represent genes upregulated in IPSC, ESC and ECC groups located between 5.65 and 0.69 radians.

The location of the specific signature genes along the PC1 and PC3 axis indicates that there is a clear classification or grouping difference among the PGC, EGC, and ESC-like stem cell groups. EGC signature genes are located in the upper half of the PC3 axis and includes genes *SRY (sex determining region Y)-box 9* (***SOX9*** *), Fibronectin 1 (* ***FN1*** *), FK506 binding protein 6 (* ***FKBP6***), *Kruppel-like Factor 4* (***KLF4***) and *AXL receptor tyrosine kinase (* ***AXL*** *)* which are significantly up-regulated in these cells. These genes may contribute to the partially reprogrammed state of EGCs in culture. PGC signature genes located in the lower half of the PC3 axis include *hemoglobin alpha 1* (***HBA1*** *)*, *X (inactive)-specific transcript* (***XIST*** *)*, *testis expressed 13A* (***TEX13*** *)*, and *SPO11 meiotic protein* (***SPO11*** *)*. *Spo11*, *Hba1* and *Tex13* have been identified in mouse microarrays as germ cell markers [31]. *XIST* is known to be down regulated in pluripotent stem cells compared to PGCs [46]. *XIST* expression was exclusively expressed by the female PGCs as expected and represents the more differentiated state of these unipotent progenitors compared to the female EGC and iPSC which also included.

Genes with shared expression levels in PGCs and EGCs were also identified and may represent a common germ cell progenitor phenotype. For example, located in the middle of the PC3 axis, commonly enriched genes in EGCs and PGCs included *imprinted maternally expressed transcript protein* (***H19*** *)*, *transforming growth factor beta 1* (***TGFB1*** *)*, *integrin alpha 8* (***ITGA8***), *insulin-like growth factor binding protein 5* (***IGFBP5***), *biglycan* (***BGN*** *)*, and *Moloney leukemia virus 10-like 1 homolog (* ***MOV10L1*** *)*. Genes that are associated with the PC1 axis define unique differences in pluripotency between PGC, EGC, and the primed human ESC-like groups. This is shown by the distinct localization of known pluripotent stem cell signature genes including *NANOG*, *MYCN*, *SOX2*, *OCT4*, and *GDF3* in the region containing ECCs, ESCs, and IPSCs.

In summary, PCA demonstrates that EGCs exhibit a unique genetic signature from PGCs and other pluripotent stem cells suggesting that EGCs represent a distinctive pluripotent state with many shared features of ESCs/IPSCs and ECCs. Moreover, these results reveal a unique set of genes which may be associated with the pluripotent state of EGCs. Thus, it is possible that these genes are uniquely turned on during the EGC stage of pluripotency and then turned off toward a more naïve like state. Consequently, it would be interesting to compare human EGCs with partially reprogrammed IPSCs and human IPSCs and ESCs to provide further insight into this issue.

### Differences in Gene Expression Profiles between PGCs and Pluripotent Stem Cell Lines Define Unique Genetic Signatures of Developmental Potency

Expression analysis of PGCs with the pluripotent stem cell lines indicated that the expression profiles of PGCs and EGCs were significantly different from the ESC, IPSC and ECC groups. The number of differentially expressed genes in EGCs, ESCs, IPSCs, and ECCs compared with PGCs are shown in **Table 2** and the complete list of genes compiled in **Table S5**. PGCs showed higher expression of 105 genes and significantly lower expression of 656 genes when compared to ESCs (**Tables S5, Sheet 1 and 2, respectively)**. Similar numbers of genes were also distinguished when PGC gene expression was compared to either IPSCs or ECCs. For instance, when PGC and ECC profiles were compared 132 genes were upregulated in PGCs compared to the up-regulation of 716 genes in ECCs (**Tables S5, Sheet 7 and 8, respectively)**. Similarly, when PGC profiles were compared to IPSCs, PGCs showed higher expression of 233 genes and decreased expression of 496 genes (**Tables S5, Sheet 5 and 6, respectively)**. Together, this data shows that a significantly higher number of genes are upregulated in the ESC, IPSC, and ECC lines compared to PGCs. Upregulation of these genes may be relevant to maintaining a primed hESC-like pluripotent state in these lines. In contrast, fewer genes were upregulated in EGCs compared to PGCs. (194 upregulated versus 94 down regulated genes) (**Tables** **S5, Sheet 3 and 4, respectively)**. Thus, more genes were altered in PGCs during their reprogramming into IPSCs, ESCs, and ECCs than EGCs potentially signifying a unique developmental state for human EGCs.

**Table 2:** Classification of genes differentially expressed in ESCs, EGCs, ECCs, and IPSCs versus PGCs.

Class	Number of Genes	Explanation	Statistics
**PGC vs. ESC**			
	105	Genes up-regulated in PGCs	FC>2, FDR adjusted p<0.0001
	656	Genes up-regulated in ESCs	FC>2, FDR adjusted p<0.0001
**PGC vs. EGC**			
	194	Genes up-regulated in PGCs	FC>2, FDR adjusted p<0.0001
	78	Genes up-regulated in EGCs	FC>2, FDR adjusted p<0.0001
**PGC vs. IPSC**			
	233	Genes up-regulated in PGCs	FC>2, FDR adjusted p<0.0001
	496	Genes up-regulated in IPSCs	FC>2, FDR adjusted p<0.0001
**PGC vs. ECC**			
	132	Genes up-regulated in PGCs	FC>2, FDR adjusted p<0.0001
	716	Genes up-regulated in ECCs	FC>2, FDR adjusted p<0.0001
**PGC vs. (ESC+IPSC+ECC)**			
	70	Genes up-regulated in PGCs	FC>2, FDR adjusted p<0.0001
	332	Genes up-regulated in (ESC+IPSC+ECC)	FC>2, FDR adjusted p<0.0001
**PGC vs. (ESC+IPSC+EGC+ECC)**			
	49	Genes up-regulated in PGCs	FC>2, FDR adjusted p<0.0001
	20	Genes up-regulated in (ESC+IPSC+EGC+ECC)	FC>2, FDR adjusted p<0.0001

Specifically, when PGCs are compared to all lines including EGCs, the number of differentially expressed genes is reduced approximately 15 fold. In this case, 20 genes were detected that were upregulated in all four stem cell lines compared to PGCs versus 332 genes in ESCs, IPSCs, and ECCs with EGCs excluded (**Tables S5, Sheet 10 and 12, respectively)**. Likewise, fewer genes are also found upregulated in PGCs when EGC profiles were combined with the analysis, i.e. 49 genes are upregulated in PGCs compared to all pluripotent lines compared to the 70 genes upregulated in PGCs when EGCs are not included (**Tables S5, Sheet 9 and 11, respectively**). Therefore, the comparisons with EGCs identified a reduced number of candidate genes which may be associated with the conversion of PGCs into the pluripotent state.

Notably, when PGCs were compared to all lines including EGCs, 20 novel associated genes were discovered that were up regulated in the stem cell lines. These included *Importin 7* (***IPO7***), *mediator complex subunit 7* (***MED7***), *RNA binding motif protein 26* (***RBM26***), and *heat shock 60 kDa protein 1* (***HSPD*** **1**). In addition, multiple genes with known or suspected roles in self-renewal or “stemcellness” were highly-up-regulated in ESCs, IPSCs, and ECCs compared to human PGCs. These included *SOX2, cyclin E1, (* ***CCNE1*** *), cyclin B1 (* ***CCNB1*** *),* and *cyclin dependent kinase* 6 (***CDK6*** *)*.

### Signature Genes Demonstrate Unique Trends in their Expression Pattern Across Lines

**Figure 4:**
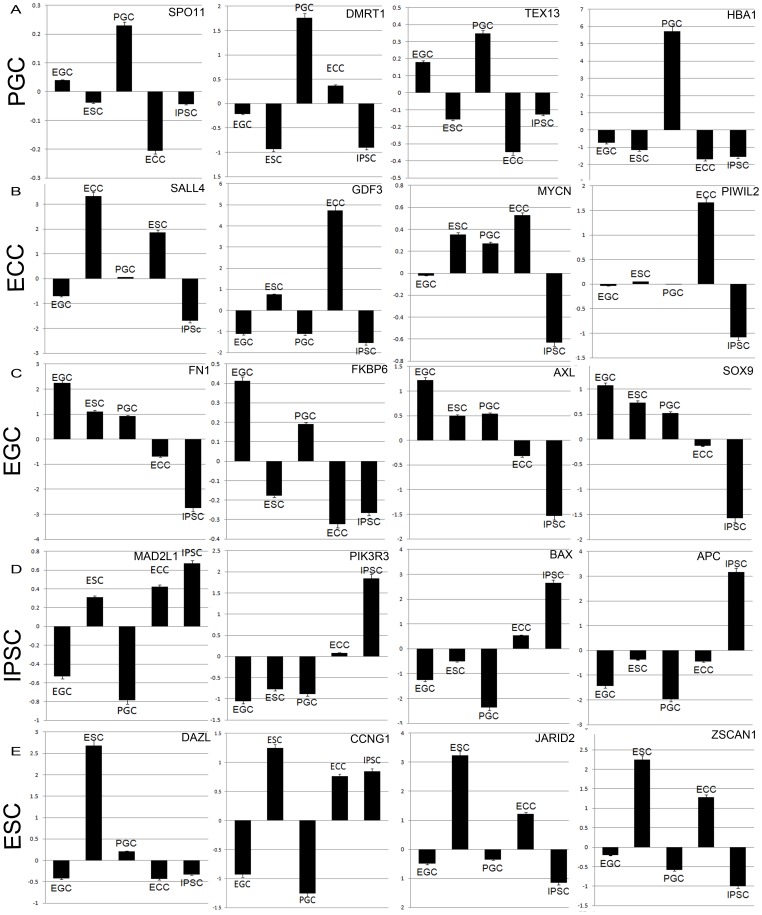
Identification of potential signature genes of PGCs, ECCs, EGCs, IPSCs, and ESCs. The vertical axis of each graph shows the log ratio of the expression data relative to the population mean. (A) Genes that are up-regulated in PGCs include (A) *SPO11, DMRT1, TEX13, and HBA1.* (B) Genes up-regulated in ECCs include *SALL4, GDF3, MYCN, and PIWIL2.* (C) Genes up-regulated in EGCs include *FN1, FKBP6, AXL, and SOX9*. (D) Genes up-regulated in IPSCs include *MAD2L1, PIK3R3, BAX and APC* **.** (E) Genes up-regulated in ESCs include *DAZL, CCNG1, JARID2, and ZSCAN1.*

Expression patterns of representative genes identified by the PCA as significantly upregulated are shown in **Figure 4**. PGC signature genes included *HBA1*, *DMRT1*, *TEX13*, and *meiotic protein SPO11*. PGCs do not form tumors while ECCs form malignant carcinomas, IPSCs form benign teratomas, and human EGCs form cyst like structures, not teratomas. Therefore, differential upregulation of expressed genes in ECCs which include *sal-like 4* (***SALL4***), *growth differentiation factor 3* (***GDF3***), *v-myc myelocytomatosis viral related oncogene*, *neuroblastoma derived* (***MYCN***), and *piwi-like 2* (***PIWIL2***) may be indicative of their oncogenic as well as their pluripotent properties in these stem cells. Indeed, all four genes have been associated with tumorigenicity, and they demonstrate a similar pattern of expression that was lowest in EGCs and IPSCs compared to ESCs.

In comparison, genes identified in the top 1000 differentially expressed genes transcripts of EGCs included *FN1, FKBP6, AXL, and SOX9*. *FN1, AXL,* and *SOX9* demonstrated a similar pattern of expression across all lines and have been shown by others to be over expressed in germ cell tumors [47]–[50]. In contrast, *FKBP6* demonstrated a different pattern in which it was uniquely upregulated in EGCs and PGCs but down regulated in ESCs, IPSCs, and ECCs. In mouse studies, this marker was significantly down regulated in both germline stem cells and ESCs compared to mouse PGCs, and therefore concluded to be a unipotent progenitor marker of mouse PGCs. Although this trend was similar in most of the human cell lines studied here, *FKBP6* was also elevated in EGCs. Thus, it is uncertain whether the difference between these two studies is due to speciation or whether up regulation of *FKBP6* is uniquely upregulated in EGCs compared to human germ line stem cells.

Interestingly, genes up regulated in IPSCs included both proliferative and anti-proliferative responses controlling cell cycle progression. For instance, IPSCs like other pluripotent stem cells expressed facilitators of the cell cycle such as *CCNE1*, *CCNB1*, *CDK6* and *CDK1*. However, IPSCs distinguished themselves from ESCs and ECCs by their elevated expression of known anti-proliferative regulators *phosphatidylinositol 3'-kinase receptor 3 (* ***PIK3R3*** *), BCL2-associated X protein (* ***BAX*** *) MAD2 mitotic arrest deficient-like 1* (***MAD2L1),*** and *adenomatous polyposis coli (* ***APC*** *)*. Previous reports have also shown similar results in IPSCs generated from somatic cells with up-regulation of these and other anti-proliferative factors involved in apoptosis, senescence and/or cell-cycle arrest (reviewed in [51]). Similar to these studies, the IPSC profiles here showed up-regulation of the apoptotic mediated factor, *BAX*, and G1 cell cycle arrest facilitator, *P16INK4a* (or ***CDKN2a***). They also expressed elevated levels of *PIK3R3* which is a member of the PI3K family and interacts with retinoblastoma protein to regulate cell proliferation and cell cycle progression. As such over expression of *PIK3R3* has been associated with ovarian, liver, prostate and breast cancers [52]. When mutated, the tumor suppressor gene, *APC,* is associated with chromosome instability and tumor progression via beta-catenin signaling [53], [54], and when normally expressed is a critical component of cellular defense mechanisms involving cell cycle arrest, DNA damage, and repair or by inducing apoptosis [55]–[57]. Together, these results support evidence in IPSCs derived from somatic cells, which also demonstrate elevated expression of anti-proliferative regulators such as these. Thus, it would be interesting to validate these results and determine whether these factors are involved in the unique signature of IPSCs as a response of their derivation or a necessary requirement of their artificially induced pluripotent state.

Genes up-regulated in ESCs include several interesting targets that have shown some implications of regulating pluripotency in mouse cells. These are shown in Figure 4 which includes deleted in azoospermia-like (***DAZL***), *CYCLIN G1 (* ***CCNG1*** *)*, AT rich interactive domain 2 *(* ***JARID2***), and zinc finger and SCAN domain containing 1 (***ZSCAN1***). *DAZL* has been identified as an early germ cell marker supporting the notion of a germ cell origin for ESCs. In the same family as *ZSCAN1*, *ZSCAN4* has been shown to be critical in regulating telomerase activity and pluripotency of mouse ESCs [58]. *CYCLIN G1* has roles in cell cycle progression and has been shown to be elevated in mouse EGCs and ESCs [59]. More recently, JARID2 has been shown to be elevated in mouse ESC and modulate pluripotency via polycomb regulation [60].

### Comparing Gene Expression Patterns in PGCs, EGCs and Pluripotent Stem Cells using Hierarchical Clustering

**Figure 5:**
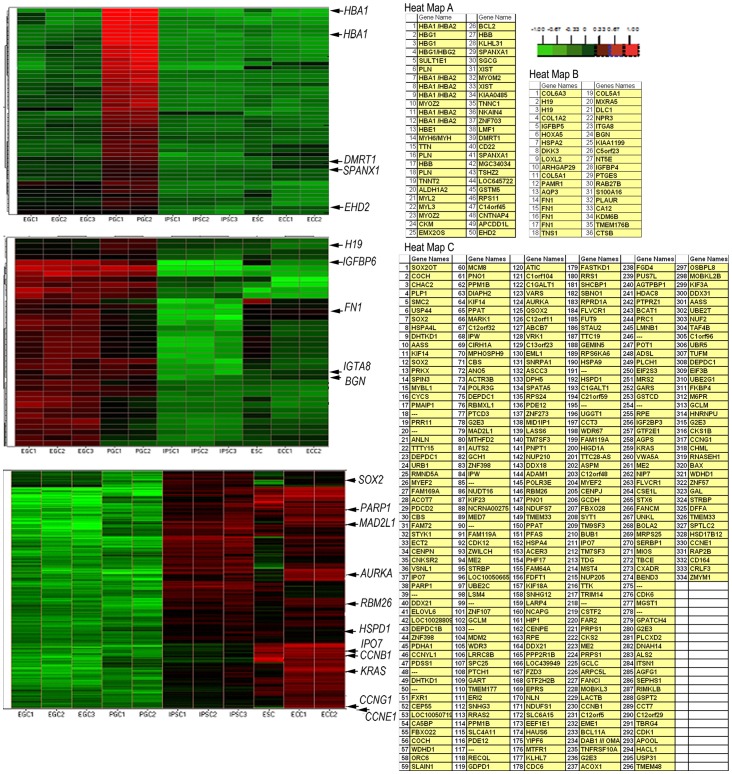
Hierarchical clustering of potential signature genes. Gene expression levels of (A) PGC signature, (B) EGC and common PGC/EGC signature and (C) ESC, IPSC and ECC group genes are represented in a heat map. Lists of genes corresponding to each group are on the right hand side of the cluster tree. Order of the genes in the tables corresponds to their order in the heat map (high expression in red, log^10^ = >1.00; low expression in green; log^10^ = <–1.00).

To further examine the relationship between human PGCs and EGCs to other pluripotent stem cell lines, signature genes were hierarchically clustered to determine their relationship across all cell lines. Hierarchical analyses was performed for genes that demonstrated the highest differential expression in pluripotent stem cells lines compared to PGCs. Approximately 60% of these genes comprised three distinct regions of gene clusters that were indicative of the PGC, EGC, or the ESC, IPSC and ECC groups. The heat maps of these regions are shown in **Figure 5**. Consistent with the PCA results, the highest levels of gene expression in PGCs compared to the pluripotent stem cells consisted of a tight cluster of several potential PGC signature genes. These genes corresponded with the loading scatter plot results and are shown to be up-regulated in **Figure 5A** including the *HBA1*, *Doublesex and mab-3 related transcription factor 1* (***DMRT1*** *)*, and *Sperm protein associated with the nucleus, X-linked, family member A1* (***SPANXA1***). These predicted signature PGC genes were also identified in the top 1000 differentially expressed genes from PCA. A different pattern between EGCs and PGCs is seen in **Figure 5B** where many of the genes that are highly up-regulated in EGCs compared to the ESC, IPSC, and ECC group were also elevated in PGCs, though not as high. These included *H19, IGFBP5, ITGA8, BGN* and *EH-domain containing 2 protein* (***EHD2***). These results further suggest that EGCs still retain marks of their unipotent PGC-like state. In contrast, *KLF4, SOX9, AXL,* and *FN1* were uniquely upregulated in EGCs compared to PGCs suggesting their involvement in EGC derivation. Similar up regulation of *KLF4* expression has been demonstrated in the conversion of mouse PGCs into EGCs [31].

Cluster analyses also revealed the overall expression patterns of potential pluripotent genes elevated in the ESCs, ECCs, and IPSCs compared to PGC and EGCs **(** **Figure 5C** **)**. As expected, *SOX2* was significantly elevated in the pluripotent stem cells compared to PGCs consistent with results from PCA. Other factors that were elevated in ESCs, ECCs, IPSCs included those involved in cell cycle namely *CCNB1, CCNE1, CDK1, CDK6, HSPD1,* ), v-Ki-ras2 Kirsten rat sarcoma viral oncogene homolog (***KRAS***) as well as those defined by the PCA including *IPO7, MED7,* and *RBM26* (**Table S4**).

### Gene Classification and Biosystems Modeling of Potential Pluripotent Pathways

**Figure 6:**
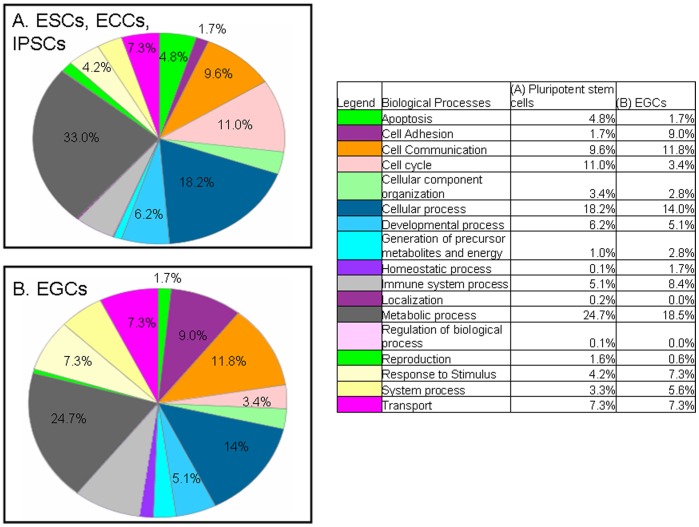
Classification of genes by specific molecular and biological function. (A) Biological processes up-regulated in ESCs, IPSCs, and ECCs compared to PGCs. (B) Biological processes up-regulated in EGCs compared to PGCs. The allocation of specific biological functions in pluripotent stem cells and EGCs are represented by percentages in the table legend.

To identify potential pathways and interpret gene relationships distinguishing EGCs from ESCs, IPSCs and ECCs, a gene classification approach was used by employing the Panther Classification System and Ingenuity Pathways Analysis (IPA). Gene classifications were performed using the Panther Classification System, and IPA was used to detect molecular connections between genes that were differentially expressed in pluripotent stem cells compared to PGCs. Together these analyses aid in the understanding of known genes in the context of biological pathways, functions and cellular processes that distinguish EGCs and PGCs from pluripotent stem cells. GO analysis shows the distribution of different cell functions, as percentages, that are allocated in ESCs, IPSCs and ECCs compared to EGCs (**Figure 6**). Distinct differences between the allocations of biological functions in EGCs compared to these stem cells are found. One of the major differences is their commitment to the cell cycle and metabolic processes. ESCs, IPSCs and ECCs allocate 11% of their functioning toward cell cycle and 33% to metabolic processes while EGCs allot 3.4% to cell cycle and 24.7% to metabolic processes. This is consistent with their reduced proliferation rates in culture. Additionally, there is a distinct difference in the percentage effort allocated for apoptotic functions in EGCs. Specifically, EGCs commit a significantly lower portion, 1.7%, of their cellular functions for regulating apoptosis, compared to ESCs, IPSCs and ECCs which allocate 4.8% percent for that same function. Taken together, these results suggest a strong role for cell cycle and apoptotic factors in maintaining a balance in stem cell self-renewal capacities. Another large difference exists between the commitments of these cells to adhesion processes. EGCs seem to allocate a larger percentage of their cellular demand (9%) to functions relating to cell adhesion while ESCs, IPSCs and ECCs allocate only 1.7%. This is consistent with a primary difficulty in maintaining EGCs in culture which includes their resilience to dispersion during subculture compared to other pluripotent stem cells. Despite the clear differences in allocation of biological processes existing between EGCs and pluripotent stem cells, there are also many similarities. For example, both cells express nearly identical percentages for transport functions, developmental processes, and cellular component organization.

**Figure 7:**
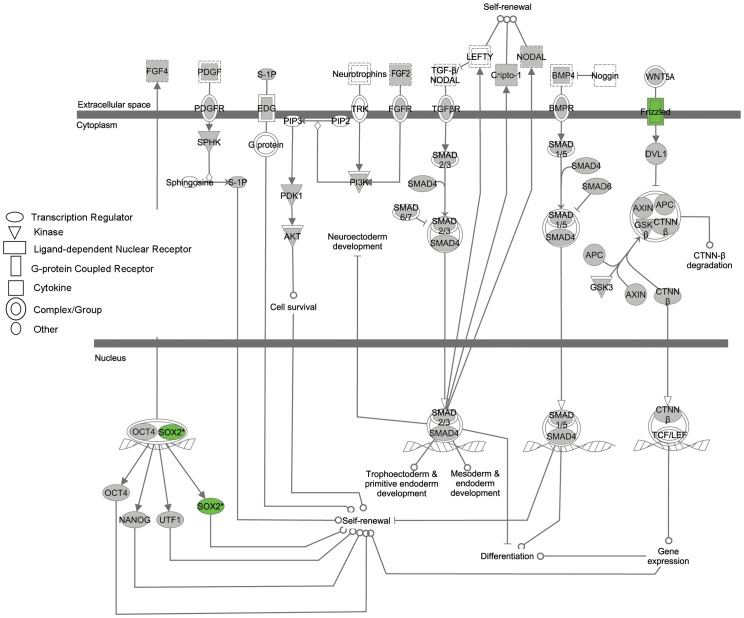
Graphical representation of biological relationships in genes responsible for human embryonic stem cell pluripotency detected by IPA analysis. Green color represents genes of the pathway that are up-regulated in the pluripotent stem cells compared to PGCs and gray color represents genes that are expressed at similar levels between both groups. White signifies genes whose expression was not detected in the cell lines.

**Figure 8:**
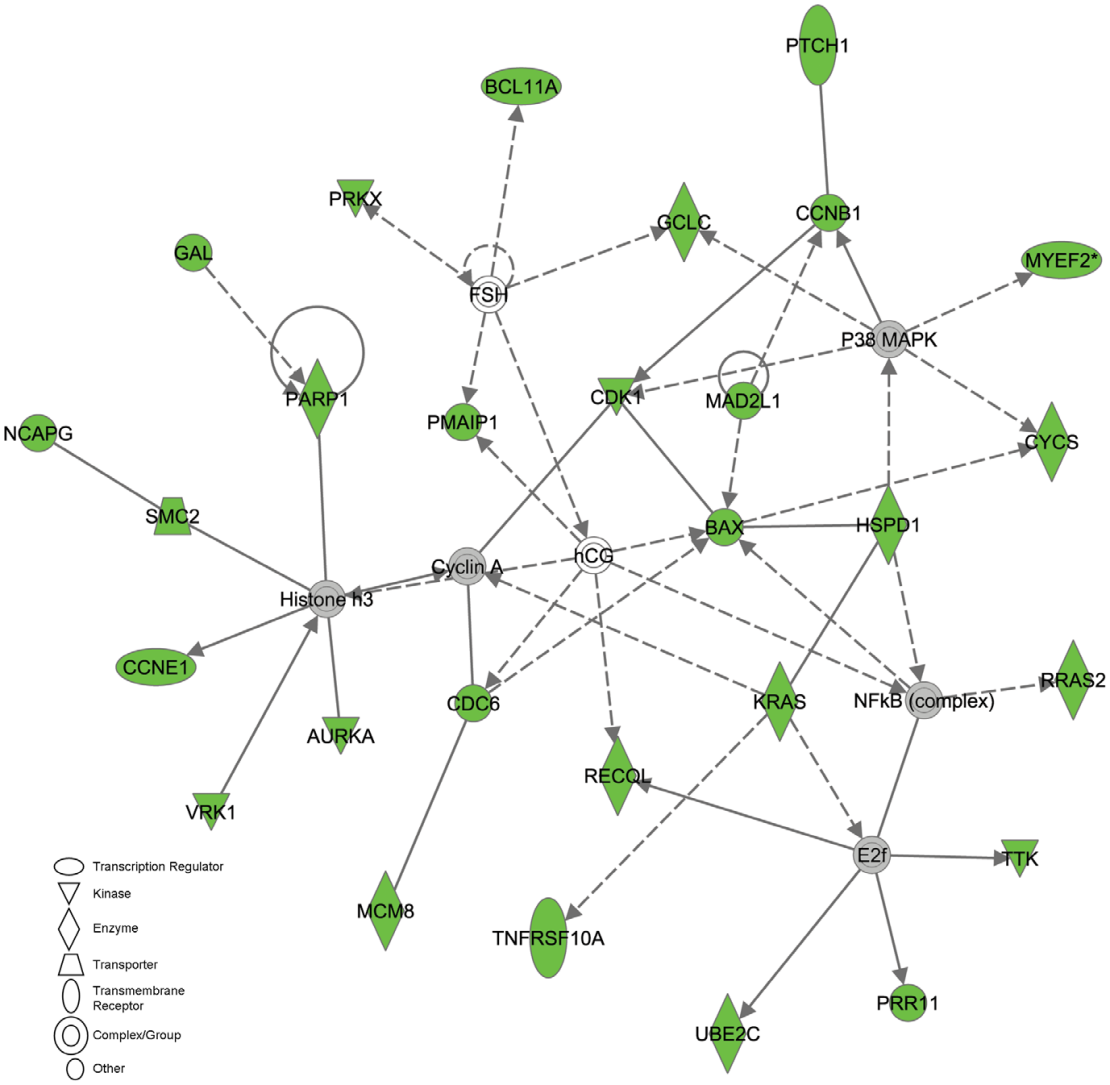
Graphical representation of biological relationships in known or suspected genes associated with controlling cell cycle, replication, DNA repair, recombination, and cell death. This network is specifically showing genes that are up-regulated in pluripotent stem cells compared to PGCs. Green color represents genes in this network that are highly up-regulated in the ESC, IPSC, and ECC group and gray color represents genes that are expressed in similar levels across all cell types. White signifies that the gene was not detected in the cell lines. Solid and dotted arrows represent direct and indirect interactions, respectively. Elevated levels of *KRAS* and *HSPD1* were also detected in EGCs.

IPA software was also used to detect potential pathways that were specifically up-regulated in pluripotent stem cells and down regulated in PGCs. **Figure 7** represents the biological relationships that exist between the regulated proteins responsible for pluripotency. Results showed that as expected the majority of these genes were expressed at similar levels (gray) among pluripotent stem cells and PGCs while *SOX2* and *FRIZZLED* were uniquely up regulated in EGCs, ESCs, IPSCs, and ECCs compared to PGCs. Other known pluripotent regulators, *NANOG* and *OCT4*, were also shown to be expressed but at similar levels among groups. Most interestingly, when genes upregulated in the pluripotent stem cells compared to PGCs was analyzed, IPA analysis identified an integral network that appears to be upregulated in pluripotent stem cells. **Figure 8** is a graphical representation of intersecting networks responsible for controlling cell cycle, DNA replication, DNA repair, recombination, and cell death in which the majority of these components were upregulated in the ESC, IPSC and ECC group compared to PGCs. This included cyclins *CCNE1, CCNG1* and *CCNB1* as well as apoptotic and DNA repair-recombination regulators, *BAX* and Poly (ADP-ribose) polymerase 1 (***PARP1***).

When EGCs were included in the comparisons, two genes with ties in cell cycle regulation, *KRAS* and *HSPD1,* were upregulated in all stem cells compared to PGCs. *KRAS* is a GTPase in the *Ras* family and is essential in normal tissue signaling of PI3–kinase but elevated in many cancers where it suppresses tumor suppressor genes. More recently, *KRAS* has also been associated with the undifferentiated state of pancreatic cancer stem cells [61] and in testicular germ cell tumors [62]. Like *KRAS, HSPD1* plays active roles in cell signaling processes and has been associated with tumorigenesis. This heat shock protein is also associated with regeneration in lower vertebrates and recently shown to be controlled by two known regulators of pluripotency, LIN28 and LET-7, (for review [63]) during retinal regeneration in zebrafish [64]. Proteomic analyses have also detected elevated expression of *HSPD1* in mouse and human ESCs [65]–[69].

## Discussion

This study is the first to report a comparative analysis of human EGCs and PGCs with other pluripotent human stem cells. These results demonstrate a unique pattern of expression for human EGCs that is distinct from PGCs and other pluripotent stem cells. Differences are also seen between human PGCs and EGCs with their mouse counterparts. Specifically, this study identified 20 novel genes that were upregulated in all pluripotent stem cells compared to PGCs and 78 genes upregulated specifically in EGCs. These novel genes provide evidence for unique factors in germ cells that may contribute to their developmental state and potentially to the intermediate pluripotent state of EGCs. These genes comprised a significant portion of regulators of the cell cycle, DNA repair, and DNA recombination. This was supported by GO analysis which demonstrated a substantial amount of energy exerted in cell cycle and metabolic processes in the stem cell lines compared to PGCs and by elevated utilization of these processes in the ESCs, IPSCs, and ECCs compared to EGCs.

### Unique Features of the PGC and EGC Transcriptional Program

Despite being committed to a single lineage, PGCs are unique because they co-express many key pluripotent genes. Specifically, we show here for the first time that human PGCs and EGCs show quantitative differences in their expression of pluripotency associated genes, *SOX2* and *DNMT3B,* compared to other human pluripotent stem cells. *SOX2,* together with *OCT4* and *NANOG*, is required for ESC cell self-renewal and pluripotency (reviewed in [70], [71]) and for reprogramming somatic cells into IPSC cells [72]. Others have similarly reported that SOX2 protein is not expressed in human PGCs [45]. Specifically, our data shows that human PGCs express *SOX2* transcript albeit at reduced levels (similar to fibroblast cell lines) while it is significantly elevated in human EGCs, IPSCs, ECCs and ESCs.

In contrast, *DNMT3B* levels in PGCs and EGCs were similar to fibroblasts compared to elevated levels in the ESC, IPSC, and ECC lines. *DNMT3B* levels are critical for DNA methylation and its expression elevated in human ESCs [34], [73]. *DNMT3B* has also been identified as one of three genes critical to distinguishing the fully reprogrammed state of pluripotent human ESCs and IPSCs [74]. Here, we show for the first time considerably lower expression of this gene in human PGCs and EGCs compared to levels detected in ESCs, IPSCs, and ECCs further suggesting that human EGCs exist in a unique pluripotent state and that reduced DNMT3B expression may be a contributing factor for the unique chromatin state of EGCs [75].

In addition to known markers of pluripotency, the analyses performed here reveal unique factors of the germ cell lineage that may also define the developmental potency of human PGCs and EGCs. Specifically, this study found several genes that were highly expressed in either human PGCs exclusively or also in EGCs compared to other pluripotent stem cells suggesting their role in regulating the unique states of these cell types. Genes upregulated in PGCs alone included *HBA1, SPANXA1*, and *DMRT1* while genes upregulated in both PGCs and EGCs included *EHD2*, *ITGA8, epithelial membrane protein 1 (* ***EMP1*** *)*, and collagen, type I, alpha 2 (***COL1A2*** *)*. These genes are interesting candidates for future studies as other reports have either directly or indirectly associated their elevated expression with the germ cell lineage yet none have reported their role in regulating germline developmental potency. For instance, *DMRT1*, up regulated in human PGCs, is a tumor suppressor gene with putative roles in regulating PGC proliferation. The absence of *Dmrt1* causes mice spermatogonia to precociously exit the spermatogonial program and enter meiosis [76]. It has also been shown to directly repress *Sox2* expression in mice and when down regulated increase teratoma formation. Reduced expression has also been identified in human germ cell tumors [77]. Thus, *DMRT1* is a potential candidate for repressing *SOX2* expression in human PGCs. Likewise, *EHD2, ITGA8* and *EMP1* has also been identified as tumor suppressor genes that when altered is associated with highly malignant ovarian cancers and germ cell tumors [62], [78]–[81]. Likewise, we show elevated expression of *COL1A2* in human PGCs and EGCs similar to others who have shown that COL1A2 distinguishes type A spermatogonia stem cells from differentiated germ cells in mouse [82]. Thus, genes found upregulated exclusively in PGCs in this study are potential candidates involved in maintaining the unipotent state of pre-meiotic, proliferative human PGCs while those upregulated in PGCs and EGCs may play a role in maintaining a unique pluripotent state of human EGCs.

Other genes that were among the most highly upregulated in PGCs included 3 of 11 genes recently identified that distinguished mouse PGCs from pluripotent germline stem cells. These genes included germ cell specific proteins like *TEX13* and meiotic protein *SPO11*, as well as the hemoglobin protein, *HBA1* [31]. Three other markers that were discussed in the report including, *Pik3r3, Mov10l1,* and *Fkbp6,* were also differentially expressed in our human cell populations. However, unlike mouse PGCs, where these genes were upregulated, in human PGCs *PIK3R3* and *FKBP6* expression was consistently down regulated compared to human stem cells, and *MOV10L1* was expressed at similar levels between human PGCs and EGCs. While these results suggest specie differences in their expression in human and mouse cells, future confirmation is warranted.

### Unique Features of the Stem Cell Transcriptional Program

From the top most differentially expressed genes, several candidates that may play a role in controlling pluripotency were discovered. These genes were highly up regulated in EGCs, IPSCs, ECCs and ESCs compared to PGCs and included genes involved in chromatin remodeling such as *IPO7*, *MED7*, *and RBM26* as well as DNA repair and transcriptional activation such as *HSPD1* and *KRAS*. These candidates are particularly interesting as recent evidence has shown roles for these factors in mechanisms that facilitate pluripotency. For instance, one report has suggested an important role of *IPO7* in the nuclear import of Sox2 and high mobility group (Hmg) box domain proteins in mouse ESCs [83] which are two well-known regulators of stem cell self-renewal. Another interesting candidate that is upregulated in EGCs compared to PGCs was *MED7*. Mediator proteins, like *MED7*, are known traditionally for their role as transcriptional co-activators required for RNA polymerase II activity [72]. However, mutations in *MED7* have recently identified a novel role for *MED7* in directly silencing subteleromic genes and increasing telomere length and life span in *Saccharomyces cerevisiae.* This makes *MED7* an interesting candidate given that telomere activity is also known to play a significant role in regulating pluripotency.

*KRAS and HSPD1* play active roles in cell signaling processes. Here we show for the first time their potential involvement in establishing the pluripotent state of human stem cells as both genes were among a few genes that were highly differentially expressed in all stem cell lines compared to PGCs. The heat shock protein, *HSPD1,* is known as a regeneration-associated gene in lower vertebrates and recently shown to be controlled by two known regulators of pluripotency, LIN28 and LET-7 [64], in zebrafish [74]. Likewise, *KRAS* has also been shown to be repressed by LET-7 in cancer cell lines resulting in reduced radio sensitivity [84]. This role of KRAS would make it consistent with a molecule that is involved in contributing to the pluripotent stem cell phenotype as stem cells are well known for their increased sensitivity to DNA damage.

It is known that pluripotent stem cells have a distinct cell-cycle from differentiated cells. They exhibit long-term proliferative capacity by spending a proportionally shorter period of time in G1 and a proportionally longer period of time in S phase compared to adult cells [85], [86]. However, it is still unknown how these distinguishing features contribute to the pluripotent state. Here, we report for the first time differences in expression of cell cycle components between human PGCs and pluripotent germline stem cells which may implicate their role in defining the pluripotent state. These included both G1 and G2 phase mediators such as *CCNB1*, *CCNG1*, *MAD2L1*, *Aurora kinase A* (***AURKA*** *)*, *HSPD1*, and *KRAS*. For instance, *CCNE1* and *CDK6* along with G1 checkpoint mediator *MAD2L1* were upregulated in ESCs, IPSCs, and ECCs but not EGCs compared to PGCs. While a few studies have shown the role of *CYCLIN D* and *CYCLIN E* in mediating the stem cell phenotype of ESCs their role in maintaining the pluripotent state of EGCs has not been studied [87]–[90]. Likewise, a similar pattern of expression in constituents of the G2/M phase including *CYCLIN B, CYCLIN G* and *CDK1* along with G2/M checkpoint regulator *AURKA* was also revealed to be upregulated in IPSCs, ECCs and ESCs compared to EGCs. Thus, it remains unknown how differences in G1 and G2 cell cycle contribute to the unique pluripotent state of EGCs which appear more similar to PGCs in their cell cycle expression than other pluripotent stem cells.

Links between pluripotency and self-renewal have been established with examples of pluripotency associated genes modulating key regulators of cell cycle and vice versa. For example, S*OX2(S)* and *OCT4 (O)* through microRNAs, and *NANOG* (N) has been shown to regulate G1 progression at the transcription level [84]. Quantitative RT-PCR analysis performed in this study specifically showed that human EGCs expressed intermediate levels of these pluripotent genes and that *SOX2* was one of the top down regulated genes in PGCs. Therefore, discovering potential links between these three genes and how they regulate the cell cycle may unravel key mechanisms involved in germ cell reprogramming. Interestingly, two cell cycle regulators potentially involved in the SON network *MYCN* and *CYCLIN E* which have established roles in defining pluripotency in human stem cells and which were down regulated in EGCs compared to other human stem cell lines [91] are likely targets for contributing to the EGC’s unique pluripotent state.

Important to the self-renewal of pluripotent stem cells is the sensitivity of these cells to DNA damage. As stem cells give rise to all cell types of the embryo, mutations incurred in early development would be detrimental to an organism [11], [92]–[96]. Thus, stem cells are hypersensitive to DNA damage [97] and as a result, demonstrate more effective stress defense pathways than more differentiated cell types. Examples of these mechanisms include removing endogenous free radicals generated by their increased proliferation [97], [98] and greater efficiency in DNA damage repair after radiation compared to their differentiated counterparts [99]. Thus, pluripotent stem cells must elicit unique responses to DNA repair and recombination. Our study reports for the first time novel connections with key regulators of DNA repair and recombination in regulating the pluripotent state including *KRAS* and *PARP1*. When activated, *KRAS* suppresses DNA repair-related tumor suppressor genes involved in homologous recombination (**HRR**) such as *BRCA1*, *BRCA2*, *TP53*, and *EXO1* [100]. Specifically, it has recently been shown that HRR is the predominant DNA double-strand breaks (DSB) repair pathway in mouse ESCs with minimal contributions by nonhomologous end joining (**NHEJ**) and microhomology-mediated end joining (**MMEJ**) repair mechanisms [101]. This is consistent with NHEJ and MMEJ being more error-prone (reviewed in ref. [102]–[105]) and theoretically resulting in unsustainable mutations. In a similar fashion, elevated expression of *PARP1* in stem cells suggests that it may also be associated with pluripotency. For instance, Poly (ADP-ribose) polymerase proteins, like *PARP1* are involved in a number of cellular processes involving mainly in DNA repair and programmed cell death and comparisons performed here support data that have shown *PARP1* is involved in the *OCT4* and *SOX2* network in mouse ESCs [24], [44].

### Conclusion

In conclusion, this study is the first to compare and analyze global gene expression profiles of human EGCs and PGCs with those of embryo-derived and germ-cell derived stem cells. The results reveal distinct features regarding the transcriptional programs of these cell types. Results from this study identified sets of genes that characterize the developmental status of human EGCs and PGCs from ECCs, IPSCs, and ESCs, and serve as important genes to delineate germ cell lineages and pluripotency in human cells. Furthermore, these findings provide important data for identifying potential mechanisms required for the reprogramming of unipotent primordial germ cells into the pluripotent state. Comparisons of PGCs identified 20 genes that are specifically upregulated in all stem cell types including EGCs provides important information for distinguishing differential pluripotent states in human germ cells and possibly conventional ESCs and IPSCs as well.

### Summary

In summary, differences in the transcriptional profiles and signature genes across different stem cell types shown here are consistent with multiple states of the pluripotency. In particular, EGCs exhibit a genetic signature distinct from other pluripotent stem cells which suggests that these stem cells are in a unique pluripotent state. These factors include novel regulators of the cell cycle, DNA repair and recombination. However, it remains to be seen whether EGCs are more like naive mouse ESCs or whether they represent a partially reprogrammed IPSC-like state. Alternatively, differences in their genetic profiles would attribute to a notion that not all pluripotency-associated genes are regulated in the same way in all pluripotent stem cells, or that epistatic interactions play a significant role in the defining pathways that generate the undifferentiated state.

## Materials and Methods

### Collection of Tissue

Gonadal tissues, primordial germ cells and embryonic germ cell lines were obtained from human fetuses 8–11 weeks post fertilization as a result of termination of pregnancy, via protocols and written patient consent, approved by the Joint Committee on Clinical Investigation of the Johns Hopkins University School of Medicine. Gestational age was confirmed by anatomical markers which include limb and digit formation, crown heel and crown rump measurement as well as the first day of the last maternal menstrual cycle. Ages are discussed in terms of fetal development and not the age from the last menstrual period. Sex of the tissue was determined by gross morphological examination of the gonads and by fluorescent *in situ* hybridization of tissue connected to the gonads as previously performed [7], [24].

### Human PGC Acquisition and EGC Derivation

PGCs were isolated using magnetic cell sorting technology (MACs) and indirect labeling of cells with magnetically tagged goat anti-mouse IgM antibodies toward a mouse-anti-SSEA1 antibody (Miltenyi Biotech). Brieﬂy, gonads were minced in 1 mg/mL collagenase, incubated at 37°C for 20 min, rinsed, and incubated with SSEA1 antibody (1∶5 dilution) for 15 min on ice. Afterward, secondary antibody was applied at 1∶100 dilution for another 30 min on ice and sorted on magnetic columns as previously described [24], [106], [107]. SSEA1+ PGCs were either directly prepared for microarray analyses or used to generate EGCs. For EGC generation, SSEA1+ PGCs from a single gonad were sorted and approximately 50 cells were seeded in each of 12 wells of a 96-well plate with irradiated mouse embryonic feeder cells, SIM 6-thioguanine resistant ouabain (STO) (∼125,000 cells/well; ATCC) [108]. Media consisted of Dulbecco’s modified Eagle’s medium-199 (Invitrogen) supplemented with 20% Knockout serum (Invitrogen), 2 ng/mL FGF2 (R&D Systems), 1000 U LIF (Millipore), 10 mM forskolin, and 20 ng/ml BMP4 (R&D Systems).

### Human ESC Culture

Human ESCs from the H1 line (WiCell, Fed ID# 0043) were cultured on matrigel (BD Biosciences) in 10-cm cell culture dishes with Dulbecco’s modified Eagle’s medium/F12-Knockout serum-based media conditioned by mouse embryonic fibroblast cells (**MEFs**) (Millipore, Strain CF1) and supplemented with 4 ng/mL FGF2 as described previously [108].

### Human ECC Culture

The human embryonal carcinoma line NTERA-2 cl.D1 and Tera-2 was acquired through American Type Culture Collection (ATCC) (Virginia) and cultured on matrigel-coated plates under conditions described previously for this cell line [109].

### Human Fibroblasts

HFF-1 line was acquired by ATCC (SCRC-1041) and is a human fibroblast cell line originally derived from the foreskins of two individuals [110]. Cells were grown in similar condition as MEFs (Millipore, Strain CF1) in Dulbecco’s Modified Eagle’s Medium, DMEM199 supplemented with 15% bovine serum albumin.

### IPSC Generation and Culture

All IPSC lines were derived from PGCs obtained by different specimens. Lentiviral production were performed using Gateway®-compatible cDNAs for *SOX2, OCT4, and MYCN* (Invitrogen) inserted via recombination with LR Clonase into a modified (attR1, attR2) pLVX-Puro vector (Clontech) using the Gateway® recombination system (Invitrogen). Purified, high titer VSV-G pseudo typed lentiviral preps for expression of each transgene were prepared using standard methods. SSEA1+ PGCs were transduced with replication-defective recombinant lentivirus for 12 hours at multiplicity of infections (MOI) of 5–10 for each construct in the presence of 6 ug/ml Polybrene (Sigma).

Lentiviral expression was confirmed by qRT-PCR analysis with transgene-specific primers. Transgenes were silenced three weeks after lentiviral transfections. Pluripotency was confirmed by *OCT4, NANOG* and *SOX2* protein expression, differentiation assays and teratoma formation assays in nude mice. Cells were maintained in culture using methods described above for ESCs.

### Micro-array Analysis

To analyze gene expression profiles, the Affymetrix Human U133 Plus 2.0 GeneChip was used. RNA extraction, reverse transcription, cRNA preparation, and chip hybridization were performed according to the manufacturer’s instructions (Affymetrix, Santa Clara, CA). In brief, total RNA was extracted from cultured cells using RNeasy (Qiagen, Valencia, CA) protocols described below for RT-PCR. Five micrograms of purified RNA were then used as a template for double-stranded cDNA synthesis primed using a T7-(dT)24 oligonucleotide. Double-stranded cDNA was then used as a template for biotin-labeled cRNA preparation using T7 RNA polymerase. Biotinylated cRNA (15 µg) was fragmented at 94°C for 35 minutes (100 mM Tris-acetate pH 8.2, 500 mM potassium acetate, 150 mM magnesium-acetate), and hybridized to the Affymetrix HG U133 Plus 2.0 GeneChips containing ∼54,000 transcripts for 16 hours at 45°C with constant rotation (60 rpm). An Affymetrix Fluidics Station 450 was used to remove the non-hybridized target and to incubate with a streptavidin-phycoerythrin conjugate to stain the biotinylated cRNA. The staining was amplified using goat IgG as blocking reagent and biotinylated goat anti-streptavidin antibody, followed by a second staining step with a streptavidin-phycoerythrin conjugate. Fluorescence was detected using the Affymetrix G3000 GeneArray Scanner and image analysis of each GeneChip was done through the GeneChip Operating System 1.4 (GCOS) software from Affymetrix, using the standard default settings. Statistical analyses of microarray data were performed using a combination of bioconductor (http://www.bioconductor.org) and Partek™ software (Version 6.5) (http://www.partek.com). The raw signal values were normalized with quantile normalization method and gene level expression was summarized using RMA (Robust Multi-Array) method [111]. Principal Component Analysis (**PCA**) were performed using Partek™ software and differential gene expression were detected using bioconductor package limma [112]. The FDR adjusted p value cutoff of <0.0001 was used to obtain the lists of differentially expressed genes. Hierarchical clustering analysis was also performed using Spotfire™.

### Quantitative Real-Time RT-PCR

RNA from EGCs, PGCs, ESCs, ECCs, and IPSCs were isolated for quantitative real-time (qRT)–polymerase chain reaction (PCR) using MiniRNeasy kits (Qiagen 74124) with the RNA clean-up protocol and optional on-column DNase treatment. Complementary DNA was generated with SuperScript III First-Strand Synthesis System RT Kits, following the manufacturer’s instructions (INV18080-051). Real-time qRT-PCR analysis was performed using ABi7900HT with Taqman Assay-on-Demand designed oligonucleotides for the detection of *OCT4*, *SOX2*, *NANOG* and *DNMT3B* and each sample had a template equivalent to 5 ng of total RNA (**Table S1**). Quantitation was measured using the DDCt method and normalized to b-actin. Each primer set was tested in at least triplicate across biological replicates.

### Statistics

t-Tests were performed to evaluate the significance between two groups. Significance was accepted at p<.05.

### Gene Classification and Biosystem Modeling

The Ingenuity Pathway Analysis (IPA) program (http://www.ingenuity.com) was used for pathway and gene classification analysis of differentially expressed genes. The microarray data set was translated into HUGO gene identifiers and uploaded to the IPA system. The IPA software is a Java based online exploratory tool with a curated database for genes with millions of published literature references. The IPA database builds gene networks, pathways, and biological function clusters. IPA software uses published literature from the database to map the biological relationship of the uploaded genes. Fisher’s exact test is used to determine the probability that each biological function is due to chance alone. Scores for IPA networks are the negative logarithm of the p-values calculated. They indicate the likelihood of the focus proteins in a network being found together due to random chance. Scores of 2 or higher have at least a 99% likelihood of not being generated by chance alone. Panther Classification System was used to perform gene ontology analyses. This system uses the Gene Ontology™ (**GO**) platform to classify genes by biological process, molecular function and cell components and includes commonly used classes of protein functions many of which are not covered by other GO analyses (www.pantherdb.org).

### Data Access

Supplemental material will be provided at www.genome.org. Raw microarray CEL files will be deposited in the GEO database with accession numbers upon acceptance of the publication.

## Supporting Information

Table S1 **The real-time qRT-PCR analysis data for the detection of** ***OCT4*** **,** ***SOX2*** **,** ***NANOG*** **and** ***DNMT3B*** **.**
(XLS)

Table S2 **Details of the cells used in this study.**
(XLS)

Table S3 **Data for PCA results including log ratio values and P-values.**
(XLSB)

Table S4 **The most highly differentially expressed genes (1000 of 54,000 or 1.85% genes in the array) represented by the red circles in** **Figure 3C** **.** These genes are compared to the overall mean across all cell lines with FDR adjusted P<0.0001.
(XLSX)

Table S5 **Complete list of differentially expressed genes in EGCs, ESCs, IPSCs, and ECCs compared to PGCs.**
(XLS)

## References

[1] Dolci S, Pesce M, De Felici M (1993). Combined action of stem cell factor, leukemia inhibitory factor, and cAMP on in vitro proliferation of mouse primordial germ cells.. Mol Reprod Dev.

[2] Witschi E (1948). Migration of the Germ Cells of Human Embryos from the Yolk Sac to the Primitive Gonadal Folds. Contributions in Embryology.. Washington: Carnegie Institute.

[3] McKay DG, Hertig AT, Adams EC, Danziger S (1953). Histochemical observations on the germ cells of human embryos.. Anat Rec.

[4] Witschi E, H.G.a.S. Grady DE (1963). Embryology of the Ovary..

[5] Resnick JL, Bixler LS, Cheng L, Donovan PJ (1992). Long-term proliferation of mouse primordial germ cells in culture.. Nature.

[6] Matsui Y, Zsebo K, Hogan BL (1992). Derivation of pluripotential embryonic stem cells from murine primordial germ cells in culture.. Cell.

[7] Hiller M, Liu CF, Blumenthal PD, Gearhart J, Kerr C (2011). Bone Morphogenetic Protein 4 Mediates Human Embryonic Germ Cell Derivation.. Stem Cells Dev.

[8] Kerr CL, Shamblott MJ, Gearhart JD (2006). Pluripotent stem cells from germ cells.. Methods Enzymol.

[9] Li XH, Cong HC, Wang Z, Wu CF, Cao YL (2002). [Isolation and culture of human pluripotent embryonic germ cells].. Shi Yan Sheng Wu Xue Bao.

[10] Turnpenny L, Brickwood S, Spalluto CM, Piper K, Cameron IT (2003). Derivation of human embryonic germ cells: an alternative source of pluripotent stem cells.. Stem Cells.

[11] Park JH, Kim SJ, Lee JB, Song JM, Kim CG (2004). Establishment of a human embryonic germ cell line and comparison with mouse and human embryonic stem cells.. Mol Cells.

[12] Andrews PW (1998). Teratocarcinomas and human embryology: pluripotent human EC cell lines. Review article.. Apmis 106: 158–167; discussion 167–158.

[13] Nakagawa M, Koyanagi M, Tanabe K, Takahashi K, Ichisaka T (2007). Generation of induced pluripotent stem cells without Myc from mouse and human fibroblasts.. Nat Biotechnol.

[14] Okita K, Ichisaka T, Yamanaka S (2007). Generation of germline-competent induced pluripotent stem cells.. Nature.

[15] Park IH, Zhao R, West JA, Yabuuchi A, Huo H (2007). Reprogramming of human somatic cells to pluripotency with defined factors.. Nature.

[16] Takahashi K, Tanabe K, Ohnuki M, Narita M, Ichisaka T (2007). Induction of pluripotent stem cells from adult human fibroblasts by defined factors.. Cell.

[17] Yamanaka S (2009). Elite and stochastic models for induced pluripotent stem cell generation.. Nature.

[18] Yu J, Vodyanik MA, Smuga-Otto K, Antosiewicz-Bourget J, Frane JL (2007). Induced pluripotent stem cell lines derived from human somatic cells.. Science.

[19] Brons IG, Smithers LE, Trotter MW, Rugg-Gunn P, Sun B (2007). Derivation of pluripotent epiblast stem cells from mammalian embryos.. Nature.

[20] Guo G, Yang J, Nichols J, Hall JS, Eyres I (2009). Klf4 reverts developmentally programmed restriction of ground state pluripotency.. Development.

[21] Tesar PJ, Chenoweth JG, Brook FA, Davies TJ, Evans EP (2007). New cell lines from mouse epiblast share defining features with human embryonic stem cells.. Nature.

[22] Hanna J, Cheng AW, Saha K, Kim J, Lengner CJ (2010). Human embryonic stem cells with biological and epigenetic characteristics similar to those of mouse ESCs.. Proc Natl Acad Sci U S A.

[23] Leitch HG, Blair K, Mansfield W, Ayetey H, Humphreys P (2010). Embryonic germ cells from mice and rats exhibit properties consistent with a generic pluripotent ground state.. Development.

[24] Kerr CL, Hill CM, Blumenthal PD, Gearhart JD (2008). Expression of pluripotent stem cell markers in the human fetal testis.. Stem Cells.

[25] Zwaka TP, Thomson JA (2005). A germ cell origin of embryonic stem cells?. Development.

[26] Rossant J (2007). Stem cells: the magic brew.. Nature.

[27] Tanaka TS, Kunath T, Kimber WL, Jaradat SA, Stagg CA (2002). Gene expression profiling of embryo-derived stem cells reveals candidate genes associated with pluripotency and lineage specificity.. Genome Res.

[28] Saitou M, Barton SC, Surani MA (2002). A molecular programme for the specification of germ cell fate in mice.. Nature.

[29] Geijsen N, Horoschak M, Kim K, Gribnau J, Eggan K (2004). Derivation of embryonic germ cells and male gametes from embryonic stem cells.. Nature.

[30] Clark AT, Bodnar MS, Fox M, Rodriquez RT, Abeyta MJ (2004). Spontaneous differentiation of germ cells from human embryonic stem cells in vitro.. Hum Mol Genet.

[31] Sabour D, Arauzo-Bravo MJ, Hubner K, Ko K, Greber B (2011). Identification of genes specific to mouse primordial germ cells through dynamic global gene expression.. Hum Mol Genet.

[32] Rao RR, Calhoun JD, Qin X, Rekaya R, Clark JK (2004). Comparative transcriptional profiling of two human embryonic stem cell lines.. Biotechnol Bioeng.

[33] Abeyta MJ, Clark AT, Rodriguez RT, Bodnar MS, Pera RA (2004). Unique gene expression signatures of independently-derived human embryonic stem cell lines.. Hum Mol Genet.

[34] Richards M, Tan SP, Tan JH, Chan WK, Bongso A (2004). The transcriptome profile of human embryonic stem cells as defined by SAGE.. Stem Cells.

[35] Sato N, Sanjuan IM, Heke M, Uchida M, Naef F (2003). Molecular signature of human embryonic stem cells and its comparison with the mouse.. Dev Biol.

[36] Sperger JM, Chen X, Draper JS, Antosiewicz JE, Chon CH (2003). Gene expression patterns in human embryonic stem cells and human pluripotent germ cell tumors.. Proc Natl Acad Sci U S A.

[37] Zeng X, Miura T, Luo Y, Bhattacharya B, Condie B (2004). Properties of pluripotent human embryonic stem cells BG01 and BG02.. Stem Cells.

[38] Assou S, Lecarrour T, Tondeur S, Strom S, Gabelle A (2007). A meta-analysis of human embryonic stem cells transcriptome integrated into a web-based expression atlas.. Stem Cells.

[39] Hanna LA, Foreman RK, Tarasenko IA, Kessler DS, Labosky PA (2002). Requirement for Foxd3 in maintaining pluripotent cells of the early mouse embryo.. Genes Dev.

[40] Okumura-Nakanishi S, Saito M, Niwa H, Ishikawa F (2004). Oct-3/4 and Sox2 regulate Oct3/4 gene in ES cells.. J Biol Chem.

[41] Avilion AA, Nicolis SK, Pevny LH, Perez L, Vivian N (2003). Multipotent cell lineages in early mouse development depend on SOX2 function.. Genes Dev.

[42] Guy J, Ellis EA, Kelley K, Hope GM (1989). Spectra of G ratio, myelin sheath thickness, and axon and fiber diameter in the guinea pig optic nerve.. J Comp Neurol.

[43] Mise N, Fuchikami T, Sugimoto M, Kobayakawa S, Ike F (2008). Differences and similarities in the developmental status of embryo-derived stem cells and primordial germ cells revealed by global expression profiling.. Genes Cells.

[44] Kerr CL, Hill CM, Blumenthal PD, Gearhart JD (2008). Expression of pluripotent stem cell markers in the human fetal ovary.. Hum Reprod.

[45] Perrett RM, Turnpenny L, Eckert JJ, O’Shea M, Sonne SB (2008). The early human germ cell lineage does not express SOX2 during in vivo development or upon in vitro culture.. Biol Reprod.

[46] Silva J, Barrandon O, Nichols J, Kawaguchi J, Theunissen TW (2008). Promotion of reprogramming to ground state pluripotency by signal inhibition.. PLoS Biol.

[47] Choufani S, Shapiro JS, Susiarjo M, Butcher DT, Grafodatskaya D (2011). A novel approach identifies new differentially methylated regions (DMRs) associated with imprinted genes.. Genome Res.

[48] Juric D, Sale S, Hromas RA, Yu R, Wang Y (2005). Gene expression profiling differentiates germ cell tumors from other cancers and defines subtype-specific signatures.. Proc Natl Acad Sci U S A.

[49] Ruoslahti E, Jalanko H, Comings DE, Neville AM, Raghavan D (1981). Fibronectin from human germ-cell tumors resembles amniotic fluid fibronectin.. Int J Cancer.

[50] Malki S, Bibeau F, Notarnicola C, Roques S, Berta P (2007). Expression and biological role of the prostaglandin D synthase/SOX9 pathway in human ovarian cancer cells.. Cancer Lett.

[51] Banito A, Gil J (2010). Induced pluripotent stem cells and senescence: learning the biology to improve the technology.. EMBO Rep.

[52] Zhang L, Huang J, Yang N, Greshock J, Liang S (2007). Integrative genomic analysis of phosphatidylinositol 3′-kinase family identifies PIK3R3 as a potential therapeutic target in epithelial ovarian cancer.. Clin Cancer Res.

[53] Aoki K, Aoki M, Sugai M, Harada N, Miyoshi H (2007). Chromosomal instability by beta-catenin/TCF transcription in APC or beta-catenin mutant cells.. Oncogene.

[54] Kielman MF, Rindapaa M, Gaspar C, van Poppel N, Breukel C (2002). Apc modulates embryonic stem-cell differentiation by controlling the dosage of beta-catenin signaling.. Nat Genet.

[55] Narayan S, Jaiswal AS (1997). Activation of adenomatous polyposis coli (APC) gene expression by the DNA-alkylating agent N-methyl-N′-nitro-N-nitrosoguanidine requires p53.. J Biol Chem.

[56] Jaiswal AS, Narayan S (2011). Assembly of the base excision repair complex on abasic DNA and role of adenomatous polyposis coli on its functional activity.. Biochemistry.

[57] Jaiswal AS, Narayan S (2008). A novel function of adenomatous polyposis coli (APC) in regulating DNA repair.. Cancer Lett.

[58] Zalzman M, Falco G, Sharova LV, Nishiyama A, Thomas M (2010). Zscan4 regulates telomere elongation and genomic stability in ES cells.. Nature.

[59] Sorrentino E, Nazzicone V, Farini D, Campagnolo L, De Felici M (2007). Comparative transcript profiles of cell cycle-related genes in mouse primordial germ cells, embryonic stem cells and embryonic germ cells.. Gene Expression Patterns.

[60] Zhang Z, Jones A, Sun CW, Li C, Chang CW (2011). PRC2 complexes with JARID2, MTF2, and esPRC2p48 in ES cells to modulate ES cell pluripotency and somatic cell reprogramming.. Stem Cells.

[61] Shankar S, Nall D, Tang SN, Meeker D, Passarini J (2011). Resveratrol inhibits pancreatic cancer stem cell characteristics in human and KrasG12D transgenic mice by inhibiting pluripotency maintaining factors and epithelial-mesenchymal transition.. PLoS One.

[62] Alagaratnam S, Lind GE, Kraggerud SM, Lothe RA, Skotheim RI (2011). The testicular germ cell tumour transcriptome.. Int J Androl 34: e133–150; discussion e150–131.

[63] Viswanathan SR, Daley GQ (2010). Lin28: A microRNA regulator with a macro role.. Cell.

[64] Ramachandran R, Fausett BV, Goldman D (2010). Ascl1a regulates Muller glia dedifferentiation and retinal regeneration through a Lin-28-dependent, let-7 microRNA signalling pathway.. Nat Cell Biol.

[65] Baharvand H, Fathi A, Gourabi H, Mollamohammadi S, Salekdeh GH (2008). Identification of mouse embryonic stem cell-associated proteins.. J Proteome Res.

[66] Van Hoof D, Mummery CL, Heck AJ, Krijgsveld J (2006). Embryonic stem cell proteomics.. Expert Rev Proteomics.

[67] Van Hoof D, Passier R, Ward-Van Oostwaard D, Pinkse MW, Heck AJ (2006). A quest for human and mouse embryonic stem cell-specific proteins.. Mol Cell Proteomics.

[68] Kurisaki A, Hamazaki TS, Okabayashi K, Iida T, Nishine T (2005). Chromatin-related proteins in pluripotent mouse embryonic stem cells are downregulated after removal of leukemia inhibitory factor.. Biochem Biophys Res Commun.

[69] Nagano K, Taoka M, Yamauchi Y, Itagaki C, Shinkawa T (2005). Large-scale identification of proteins expressed in mouse embryonic stem cells.. Proteomics.

[70] Masui S, Nakatake Y, Toyooka Y, Shimosato D, Yagi R (2007). Pluripotency governed by Sox2 via regulation of Oct3/4 expression in mouse embryonic stem cells.. Nat Cell Biol.

[71] Takahashi K, Yamanaka S (2006). Induction of pluripotent stem cells from mouse embryonic and adult fibroblast cultures by defined factors.. Cell.

[72] Zhu X, Liu B, Carlsten JO, Beve J, Nystrom T (2011). Mediator influences telomeric silencing and cellular life span.. Mol Cell Biol.

[73] Gopalakrishnan S, Van Emburgh BO, Shan J, Su Z, Fields CR (2009). A novel DNMT3B splice variant expressed in tumor and pluripotent cells modulates genomic DNA methylation patterns and displays altered DNA binding.. Mol Cancer Res.

[74] Chan EM, Ratanasirintrawoot S, Park IH, Manos PD, Loh YH (2009). Live cell imaging distinguishes bona fide human iPS cells from partially reprogrammed cells.. Nat Biotechnol.

[75] Onyango P, Jiang S, Uejima H, Shamblott MJ, Gearhart JD (2002). Monoallelic expression and methylation of imprinted genes in human and mouse embryonic germ cell lineages.. Proc Natl Acad Sci U S A.

[76] Matson CK, Murphy MW, Griswold MD, Yoshida S, Bardwell VJ (2010). The mammalian doublesex homolog DMRT1 is a transcriptional gatekeeper that controls the mitosis versus meiosis decision in male germ cells.. Dev Cell.

[77] Krentz AD, Murphy MW, Kim S, Cook MS, Capel B (2009). The DM domain protein DMRT1 is a dose-sensitive regulator of fetal germ cell proliferation and pluripotency.. Proc Natl Acad Sci U S A.

[78] Cai LY, Abe M, Izumi S, Imura M, Yasugi T (2007). Identification of PRTFDC1 silencing and aberrant promoter methylation of GPR150, ITGA8 and HOXD11 in ovarian cancers.. Life Sci.

[79] Quinn MC, Filali-Mouhim A, Provencher DM, Mes-Masson AM, Tonin PN (2009). Reprogramming of the transcriptome in a novel chromosome 3 transfer tumor suppressor ovarian cancer cell line model affected molecular networks that are characteristic of ovarian cancer.. Mol Carcinog.

[80] Santin AD, Zhan F, Bellone S, Palmieri M, Cane S (2004). Gene expression profiles in primary ovarian serous papillary tumors and normal ovarian epithelium: identification of candidate molecular markers for ovarian cancer diagnosis and therapy.. Int J Cancer.

[81] Bignotti E, Tassi RA, Calza S, Ravaggi A, Romani C (2006). Differential gene expression profiles between tumor biopsies and short-term primary cultures of ovarian serous carcinomas: identification of novel molecular biomarkers for early diagnosis and therapy.. Gynecol Oncol.

[82] He Z, Feng L, Zhang X, Geng Y, Parodi DA (2005). Expression of Col1a1, Col1a2 and procollagen I in germ cells of immature and adult mouse testis.. Reproduction.

[83] Boiani M, Scholer HR (2005). Regulatory networks in embryo-derived pluripotent stem cells.. Nat Rev Mol Cell Biol.

[84] Singh AM, Dalton S (2009). The cell cycle and Myc intersect with mechanisms that regulate pluripotency and reprogramming.. Cell Stem Cell.

[85] Filipczyk AA, Laslett AL, Mummery C, Pera MF (2007). Differentiation is coupled to changes in the cell cycle regulatory apparatus of human embryonic stem cells.. Stem Cell Res.

[86] Lee NS, Kim JS, Cho WJ, Lee MR, Steiner R (2008). miR-302b maintains “stemness” of human embryonal carcinoma cells by post-transcriptional regulation of Cyclin D2 expression.. Biochem Biophys Res Commun.

[87] Zhang Z, Arora S, Zhou Y, Cherry A, Wang TS (2011). Replication-compromised cells require the mitotic checkpoint to prevent tetraploidization.. Chromosoma.

[88] Wang L, Yin F, Du Y, Chen B, Liang S (2010). Depression of MAD2 inhibits apoptosis and increases proliferation and multidrug resistance in gastric cancer cells by regulating the activation of phosphorylated survivin.. Tumour Biol.

[89] Guo Y, Zhang X, Yang M, Miao X, Shi Y (2010). Functional evaluation of missense variations in the human MAD1L1 and MAD2L1 genes and their impact on susceptibility to lung cancer.. J Med Genet.

[90] Schvartzman JM, Duijf PH, Sotillo R, Coker C, Benezra R (2011). Mad2 is a critical mediator of the chromosome instability observed upon Rb and p53 pathway inhibition.. Cancer Cell.

[91] Orford KW, Scadden DT (2008). Deconstructing stem cell self-renewal: genetic insights into cell-cycle regulation.. Nat Rev Genet.

[92] Corbet SW, Clarke AR, Gledhill S, Wyllie AH (1999). P53-dependent and -independent links between DNA-damage, apoptosis and mutation frequency in ES cells.. Oncogene.

[93] Aladjem MI, Spike BT, Rodewald LW, Hope TJ, Klemm M (1998). ES cells do not activate p53-dependent stress responses and undergo p53-independent apoptosis in response to DNA damage.. Curr Biol.

[94] Van Sloun PP, Jansen JG, Weeda G, Mullenders LH, van Zeeland AA (1999). The role of nucleotide excision repair in protecting embryonic stem cells from genotoxic effects of UV-induced DNA damage.. Nucleic Acids Res.

[95] Roos WP, Christmann M, Fraser ST, Kaina B (2007). Mouse embryonic stem cells are hypersensitive to apoptosis triggered by the DNA damage O(6)-methylguanine due to high E2F1 regulated mismatch repair.. Cell Death Differ.

[96] Filion TM, Qiao M, Ghule PN, Mandeville M, van Wijnen AJ (2009). Survival responses of human embryonic stem cells to DNA damage.. J Cell Physiol.

[97] Saretzki G, Armstrong L, Leake A, Lako M, von Zglinicki T (2004). Stress defense in murine embryonic stem cells is superior to that of various differentiated murine cells.. Stem Cells.

[98] Maynard S, Swistowska AM, Lee JW, Liu Y, Liu ST (2008). Human embryonic stem cells have enhanced repair of multiple forms of DNA damage.. Stem Cells.

[99] Tsunoda T, Takashima Y, Fujimoto T, Koyanagi M, Yoshida Y (2010). Three-dimensionally specific inhibition of DNA repair-related genes by activated KRAS in colon crypt model.. Neoplasia.

[100] Tichy ED, Pillai R, Deng L, Liang L, Tischfield J (2010). Mouse embryonic stem cells, but not somatic cells, predominantly use homologous recombination to repair double-strand DNA breaks.. Stem Cells Dev.

[101] Mao Z, Bozzella M, Seluanov A, Gorbunova V (2008). DNA repair by nonhomologous end joining and homologous recombination during cell cycle in human cells.. Cell Cycle.

[102] McVey M, Lee SE (2008). MMEJ repair of double-strand breaks (director’s cut): deleted sequences and alternative endings.. Trends Genet.

[103] Menissier de Murcia J, Ricoul M, Tartier L, Niedergang C, Huber A (2003). Functional interaction between PARP-1 and PARP-2 in chromosome stability and embryonic development in mouse.. Embo J.

[104] Gao F, Kwon SW, Zhao Y, Jin Y (2009). PARP1 poly(ADP-ribosyl)ates Sox2 to control Sox2 protein levels and FGF4 expression during embryonic stem cell differentiation.. J Biol Chem.

[105] Pardo M, Lang B, Yu L, Prosser H, Bradley A (2010). An expanded Oct4 interaction network: implications for stem cell biology, development, and disease.. Cell Stem Cell.

[106] Kerr CL, Gearhart JD, Elliott AM, Donovan PJ (2006). Embryonic germ cells: when germ cells become stem cells.. Semin Reprod Med.

[107] Hiller M, Liu CF, Blumenthal PD, Gearhart J, Kerr C (2010). Bone Morphogenetic Protein 4 Mediates Human Embryonic Germ Cell Derivation.. Stem Cells Dev.

[108] Chaerkady R, Kerr CL, Kandasamy K, Marimuthu A, Gearhart JD (2010). Comparative proteomics of human embryonic stem cells and embryonal carcinoma cells.. Proteomics.

[109] Prigione A, Hossini AM, Lichtner B, Serin A, Fauler B (2011). Mitochondrial-associated cell death mechanisms are reset to an embryonic-like state in aged donor-derived iPS cells harboring chromosomal aberrations.. PLoS One.

[110] Bolstad BM, Irizarry RA, Astrand M, Speed TP (2003). A comparison of normalization methods for high density oligonucleotide array data based on variance and bias.. Bioinformatics.

[111] Irizarry RA, Bolstad BM, Collin F, Cope LM, Hobbs B (2003). Summaries of Affymetrix GeneChip probe level data.. Nucleic Acids Res.

[112] Smyth GK (2004). Linear models and empirical bayes methods for assessing differential expression in microarray experiments.. Stat Appl Genet Mol Biol.

